# Book Reviews

**Published:** 1902-07

**Authors:** 


Simon's Clinical Diagnosis. A Manual of Clinical Diagnosis by means of Microscopical and Chemical Methods. For Students, Hospital Physicians and Practitioners. By Charles E. Simon, M. D., author of Simon's Physiological Chemistry, etc New (4th) edition, thoroughly revised and enlarged. Octavo, 608 pages, illustrated with 139 engravings and 19 plates in colors. Philadelphia and New York: Lea Brothers & Co. 1902. [Price, cloth, $3.75 net.]

Four editions of this manual have appeared since 1896, which is strong testimony as to its value and appreciation by the students and professional men, for whom it is more especially intended. During this period there has been much advance in laboratory methods of research, particularly as applied to clinical diagnosis, and the work of this author has contributed not a little toward this improvement. Moreover, there is a constantly increasing appreciation of the value of clinical diagnosis by means of the microscope and chemistry.

The methods of research by these methods are growing simpler as experience increases, so that now the general physician, however busy he may be, can apply them in a practical manner without exhausting a great deal of time. In this edition considerable new material has been incorporated, which serves to keep it abreast of the period, and references to the literature relating to this important topic have been added, all of which will serve to increase the value of the work to original worker, student and teacher. The illustrations, too, have been increased in number as well as in value, until now the manual becomes one of the foremost works of its kind, and it appeals strongly to everyone who is studying, teaching or practising this branch of medical science.

THE PRACTICAL MEDICINE SERIES OF YEAR BOOKS. Ten volumes. Issued monthly, under the general editorial charge of GUSTAVUS P. HEAD, M. D., Professor of Laryngology and Rhinology in the Chicago Post-Graduate Medical School. Vol. III. The Eye, Ear, Nose and Throat. Edited by CASEY A. WOOD, ALBERT H. ANDREWS and T. MELVILLE HARDIE. December, 1901. Pp. 346. Chicago: The Year Book Publishers. [Price, $1.50.]

In the preparation of the topics for this book that upon the eye was committed to Casey A. Wood, upon the ear to Albert H. Andrews, and upon the nose and throat to T. Melville Hardie, all of Chicago. It must be conceded that the selections are good ones, and that each has done his work with care. This series of year books, of which the one before us is the third volume, has been prepared for the special use of general practitioners of medicine. In the special branches dealt with in this number it is necessary that he must have intelligent information, though he may seldom employ it practically. It is obviously out of place to offer a critical review of the book here, but we may invite attention with propriety to that part presenting mastoid disease as being worthy of note. So many obscure symptoms arise from lesions of the middle-ear and mastoid that differentiation is often a matter of expertness, but the instructions herein presented are extremely helpful to practical physicians.

A REFERENCE HANDBOOK OF THE MEDICAL SCIENCE. Embracing the Entire Range of Scientific and Practical Medicine and Allied Science. By various writers. A new edition, completely revised and rewritten. Edited by ALBERT H. BUCK, M. D., New York City. Volume IV. Illustrated by chromolithographs and 859 half-tone and wood engravings. Nine volumes, imperial 8vo. New York: William Wood & Co. 1902. [Price, muslin, $6.00 per volume; leather, $7.00 per volume; half morocco, $8.00 per volume.]

In the editorial columns of the June edition of the JOURNAL we invited attention to this volume of the reference handbook, in commenting upon the article on examining and licensing boards, written by Mr. James Russell Parsons, Jr., of the University of the State of New York. We take pleasure in further mentioning this practical book in this place, because from several viewpoints it is the best of the first half of the set. It is, obviously, out of place to undertake a critique of the volume article by article, but we may point out, with great propriety, the fact that in this book the monograph on fractures, by Edward L. Keyes, Jr., is deserving of the closest scrutiny. It is abreast of the period in its teachings and supplies just that information the busy physician requires; besides, it is copiously illustrated with wood engravings and a colored plate that are faithful depictions of real conditions.

The article on gynecological examinations is another excellent presentation of an important subject, also well illustrated. The book is full of good material, which is well arranged and accessible.

---

A Practical Treatise on Materia Medica and Therapeutics. With Especial Reference to the Clinical Application of Drugs By John V. Shoemaker, M. D., LL. D., Professor of Materia Medica, Pharmacology, Therapeutics and Clinical Medicine, and Clinical Professor of Diseases of the Skin in the Medico-Chirurgical College of Philadelphia; Physician to the Medico Chirurgical Hospital. Fifth edition, thoroughly revised. Octavo, pages viii.—1143. Philadelphia: F .A. Davis Co. 1901. [Extra cloth, $5.00 net; sheep, $5.75 net, delivered.]

This new edition, the fifth, of this standard treatise will receive a cordial welcome from undergraduates and physicians, as have the previous issues. Therapeutics has progressed with the other departments of medicine and new editions of these works are needed at frequent intervals. The author of this book has taken advantage of his opportunity and reviewed the literature of the subjects he deals with, incorporating in his treatise such methods, drugs or chemical improvements relating to the treatment of the sick as have value.

In it, too, he has recorded the finding of new applications for some of the older articles of the materia medica, the later researches concerning the physiological action of remedies, and such new medicinal plants as have been found efficacious are described. The preparations of both the British and American pharmacopeias are given, which makes the book additionally valuable for reference. For these and other reasons that might be enumerated, this treatise has become one of the most popular of its kind.

THE MEDICAL NEWS POCKET FORMULARY. New (4th) edition. Containing 1,700 prescriptions, representing the latest and most approved methods of administering remedial agents. By E. QUIN THORNTON, M D., Demonstrator of Therapeutics, Pharmacy and Materia Medica in the Jefferson Medical College, Philadelphia. Carefully revised to date of issue. In one wallet-shaped volume, strongly bound in leather, with pocket and pencil. Philadelphia and New York: Lea Brothers & Co. 1902. [Price, $1.50 net.]

By some mischance the third edition of this excellent formulary, received over a year ago, escaped notice in these columns —a circumstance which we very much regret. Now comes the fourth edition, and we can only repeat what we have said about earlier issues—namely, that it is one of the best of its kind and excels most of them in its convenience. The descriptive heading above given is an excellent resumé of the contents and style of the book, which every physician will find extremely useful.

---

TRANSACTIONS OF THE AMERICAN LARYNGOLOGICAL ASSOCIATION at the Twenty-third Annual Meeting, held at New Haven, Conn., May 27, 28 and 29, 1901. Edited by the Secretary, JAMES E. NEWCOMB, M. D. New York: The Roony & Otten Printing Co. 1901.

As usual, the annual volume issued by this excellent society for 1901, is replete with interest. The president's address, by Dr. Henry L. Swain, presents an interesting review of the work the association has wrought, under the title, Laryngology and its place in medical education. When the association was formed the laryngologists were few and scarcely known outside the larger centers of population. Now, everybody who thinks he sees an easy road to success through this specialty, takes a six weeks' course somewhere, or with somebody, and behold! he is a laryngologist, or at least a rhinologist.

A painful reminder of the sad death of Dr. William H. Daly, about a year ago, is presented in the beautiful tribute paid to his memory through the biographic memorial written by Dr. John O. Roe, of Rochester. A few typographic errors in the book have been corrected by supplementary paster slips—a most excellent plan.

The Pocket Gray or Anatomist's Vade-Mecum. By the late Edward Cotterell, F.R.C.S. Fifth edition, revised and edited by C. H. Fogge, M.D., M. S. (London), F. K. C. S., Surgeon Demonstrator of Anatomy at Guy's Hospital. Twentieth thousand. New York: William Wood & Co. 1901. [Price, $ .]

This convenient anatomical reminder has stood the test of observation and experience running back through many years. It is always well for the undergraduate and junior physician, not to mention the more matured practitioner, to have at hand some such book to serve in the haste of study or work, when the larger volume cannot be consulted, and there is none better than this one. It is based upon Gray's Anatomy, which is a sufficient endorsement.

---

A Brief Manual of Prescription-Writing in Latin or English. For the use of Physicians, Pharmacists and Medical and Pharmacal Students. By M. L. Neff, A.M., M.D., Cedar Rapids, Ia. Pages v.—152. Size, 8 x 5¾ inches. Philadelphia: F. A. Davis Co., Publishers, 1914-16 Cherry St. [Extra cloth, 75 cents net, delivered.]

A manual of this kind is of value to the beginner, who should start right in writing prescriptions. Too often little heed is paid to correct English, not to criticise the Latin of the writer of prescriptions. A careful study of this manual will serve to aid in correcting or perfecting both defects. A number of useful tables are added to the main features of this work, and some blank pages are provided for the preservation of such formulæ as may prove of value.

---

A MANUAL OF GYNECOLOGY FOR THE USE OF STUDENTS AND GENERAL PRACTITIONERS. By F. H. DAVENPORT, A. B., M. D., Assistant Professor in Gynecology, Harvard Medical School. New (4th) edition, revised and enlarged in one 12mo volume of 402 pages with 154 illustrations. Philadelphia and New York: Lea Brothers & Co. 1902. [Price, cloth, $1.75 net.]

This superior manual, now in its fourth edition, is one of the best of the smaller works on gynecology, and in some respects excels the others. It is a true manual in size and make-up, but it also contains much that is usually reserved for the general treatise. For example, hysterectomy, the most formidable operation in the gynecological domain, is given a brief but excellent description which is well illustrated. Fibromyomata, too, are well described as indeed are some other topics that are usually found only in textbooks. The increased demand for the book, when it enlarged its scope by invading the surgical field, as it did in its third edition, gives ample testimony of its appreciation by the advanced practitioner. In this edition the same lines have been followed and its value continues to increase with each new issue. We commend it also to the student, for that part of the work which may be termed elementary is addressed more espeically to him.

---

QUIZ COMPENDS. Compend of General Pathology. By ALFRED EDWARD THAYER, M. D., Assistant Instructor in Gross Pathology, Cornell Medical College; Pathologist to the City Hospital. Containing 78 illustrations. Philadelphia: P. Blakiston's Son & Co. 1902. [Price, 80 cents net.]

If the study of pathology can be stimulated by a quiz manual such as this, then we can not only tolerate but praise the plan of teaching by quiz compends. At all events, this particular book is skilfully arranged by a practical teacher and cannot fail to receive the approbation of those for whom it is intended.

---

TRANSACTIONS OF THE STATE MEDICAL SOCIETY OF WISCONSIN. Fourth Year, 1901. Including Constitution and By-Laws. Edited by CHARLES S. SHELDON, M.D., Secretary. Madison: State Journal Printing Co. 1901.

The State of Wisconsin has reason to be proud of its medical society, judging by the excellent work it performs at its annual meetings. Its transactions compare favorably with those of older and larger states, and the volume is well edited by the accomplished secretary of the society. At the session of 1901, Dr. George M. Gould, of Philadelphia, delivered the address in medicine, choosing for his subject "Disease and sin." The book contains a number of valuable scientific communications and is better printed and bound than are many transactions' volumes.

